# The impact of a cancer diagnosis on health and well‐being: a prospective, population‐based study

**DOI:** 10.1002/pon.3998

**Published:** 2015-10-01

**Authors:** Kate Williams, Sarah E. Jackson, Rebecca J. Beeken, Andrew Steptoe, Jane Wardle

**Affiliations:** ^1^ Health Behaviour Research Centre, Department of Epidemiology and Public HealthUniversity College LondonLondonUK; ^2^ Psychobiology Group, Department of Epidemiology and Public HealthUniversity College LondonLondonUK

**Keywords:** cancer, oncology, survivorship, longitudinal, quality of life, depression

## Abstract

**Objective:**

Little is known about the trajectory of health and well‐being from before to after a cancer diagnosis. This study aimed to examine changes in health and well‐being across three time points (0–2 years before a cancer diagnosis, 0–2 years post‐diagnosis and 2–4 years post‐diagnosis) in individuals receiving a new cancer diagnosis, and at matched time points in a cancer‐free comparison group.

**Methods:**

Data were from waves 1–6 of the English Longitudinal Study of Ageing. Repeated‐measures ANOVAs were used to examine differences in self‐rated health, mobility impairments, activities of daily living impairments, quality of life, depressive symptoms and life satisfaction by group and time, and group‐by‐time interactions.

**Results:**

Of the 4565 participants with data from three time points, 444 (9.7%) reported a new cancer diagnosis. Those in the cancer group reported poorer self‐rated health (*p* < .001), quality of life (*p* < .001) and life satisfaction (*p* < .01) than participants in the comparison group, and a higher proportion reported depressive symptoms (*p* < .001) and impairments in mobility (*p* < .001) and activities of daily living (*p* < .001). All markers of health and well‐being worsened significantly over time. The group‐by‐time interaction was significant for self‐rated health (*p* < .001), with a greater decline in health over time in the cancer group.

**Conclusions:**

Cancer survivors in this sample had poorer health and well‐being than those with no diagnosis, and self‐rated health deteriorated more rapidly following a cancer diagnosis. Screening for these factors around the time of a cancer diagnosis could allow for interventions to be targeted effectively and improve the health and well‐being of cancer survivors. © 2015 The Authors. *Psycho‐Oncology* published by John Wiley & Sons Ltd.

## Background

There are now an estimated 36.2 million cancer survivors worldwide 1, 2. This has led to increasing research attention on optimising the well‐being, health and survival of those living beyond a diagnosis of cancer.

Population‐based studies have typically found that cancer survivors have poorer quality of life 3, 4, more anxiety 5 and depressed mood 4; have more limitations of activities of daily living (ADL) 6 and report poorer health 6, 7 than those without a diagnosis of cancer. They also have greater use of mental health services 8. In the UK, cancer survivors have been found to experience poorer health and well‐being across a wide range of outcomes than individuals with a chronic condition other than cancer 9.

Very few longitudinal studies have examined changes in health and well‐being following a cancer diagnosis. One compared 206 cancer survivors with 120 healthy age‐matched controls at 3 months, 15 months and 8 years post‐cancer diagnosis 10. Compared with the healthy comparison group, cancer survivors experienced more physical symptoms and these persisted at the 8‐year follow‐up. They had more depressive symptoms at 3 months, although differences were no longer significant at 15 months or 8 years post‐diagnosis. These findings suggest that some of the adverse effects may diminish over time. In contrast, another study found that 10 years post‐diagnosis, breast cancer survivors not only experienced poorer physical and social functioning than controls, but that these differences increased in the long term 11, suggesting that health and well‐being may be affected for many years after a cancer diagnosis.

None of the studies to date have included pre‐diagnosis data, making it is difficult to determine if the health and well‐being effects are a direct consequence of their cancer diagnosis. The acute impact of cancer diagnosis and treatment may cause health and well‐being to deteriorate, but some of the differences may be long‐standing. Therefore, the aim of study was to examine the trajectory of health and well‐being from pre‐cancer diagnosis to multiple times post‐diagnosis, in order to determine how health and well‐being change following a cancer diagnosis, and whether they return to pre‐diagnosis levels. Specifically, it examined the effect of a cancer diagnosis on changes in health and well‐being from 0 to 2 years before a cancer diagnosis, through 0–2 and 2–4 years after diagnosis, using data from a population‐based sample of older adults in the UK. The comparison sample comprised all people who did not receive a diagnosis of cancer, regardless of other diagnoses, in order to examine the specific influence of a cancer diagnosis, over and above the range of health conditions that affect people at older ages.

## Methods

### Design and participants

Data were from waves 1–6 of the English Longitudinal Study of Ageing (ELSA), which were collected at two yearly intervals between 2002 and 2012. Details on the cohort and sampling method have been published elsewhere 12, but briefly, ELSA is a panel study recruited from households with one or more members aged ≥50 years responding to the Health Survey for England in 1998, 1999 and 2001 (core sample: *n* = 12 099), with ‘refreshment samples’ added from additional rounds of the Health Survey for England in 2006, 2008 and 2012. Participants are followed up every 2 years, where they complete a computer‐assisted personal interview during a home visit and a self‐completed questionnaire that is returned by post. The present study uses data on health and well‐being from participants reporting a new cancer diagnosis in any of waves 2 to 5 and control participants who were cancer‐free at all six waves. ELSA has received approval from various ethics committees, including the London Multi‐Centre Research Ethics Committee, and full informed consent has been obtained from all participants.

The analysed sample included participants who reported a new cancer diagnosis in waves 2 to 5 and had provided data at both the previous and subsequent wave. The first wave in which a cancer diagnosis was reported became the ‘peri‐diagnosis’ point (T1), the previous wave was the ‘pre‐diagnosis’ point (T0) and the subsequent wave was the ‘post‐diagnosis point’ (T2). Individuals reporting a cancer diagnosis at wave 1 or a new diagnosis at wave 6 were excluded from the analysis because of the absence of pre‐diagnosis or post‐diagnosis data respectively. Likewise, participants from a refreshment cohort reporting a cancer diagnosis on joining the study were excluded for the same reason.

The comparison group comprised participants who had not received a cancer diagnosis in any wave. This was favoured over a completely healthy control group (as used in some studies) because it enabled us to determine the specific influence of a cancer diagnosis, over and above any other chronic diseases. To match the time points used for the cancer survivor group, for continuous variables, we used the mean of all possible pre‐diagnosis waves for T0 (waves 1 to 4), the mean of all possible peri‐diagnosis waves for T1 (waves 2 to 5) and the mean of all possible post‐diagnosis waves for T2 (waves 3 to 6); giving an average interval of 2 years between each time point. For categorical variables, we calculated mean scores following the same method and dichotomised these scores at the relevant cut‐point.

For both the cancer survivor and comparison groups, we only included individuals with data on at least one outcome variable at all three time points (T0, T1 and T2).

### Measures

#### Cancer diagnosis

A cancer diagnosis was defined as answering ‘yes’ to the question: ‘*Have you ever been told by a doctor or other health professional that you had cancer or any other kind of malignancy*’. If they said yes they were asked ‘*In which part of body did the cancer/cancers/malignant tumor start*’ with response options: lung/breast/colon, bowel or rectum, lymphoma/leukaemia/melanoma or other skin cancer/somewhere else. For these analyses, we included all cancers combined.

#### Demographic characteristics

Age and sex were included as control variables. Household non‐pension wealth used as an indicator of socio‐economic status, as it has been identified as particularly appropriate to this age group 13.

### Co‐morbidities

The presence of co‐morbidities was assessed with the question: ‘*Has a doctor ever told you have any of the following conditions…coronary heart disease (CHD), diabetes, arthritis, asthma, stroke, chronic lung disease and hypertension*’ [select all that apply].

#### Health and quality of life

Self‐rated health was assessed using a single item: ‘*Would you say your health is… poor/fair/good/very good/excellent?*’ Scores ranged from 1 to 5 with higher scores indicating better self‐rated health.

Mobility was assessed by asking participants if they had any difficulty performing 10 everyday activities because of a health problem (excluding any that they expected to last less than three months). Activities included walking 100 yards, sitting for about 2 h and getting up from a chair after sitting for long periods. A binary (yes/no) response format was used, with scores ranging from 0 to 10 according to the number of activities with which a difficulty was reported. Because the resulting summary scores were highly skewed, with most participants reporting no difficulty performing any activities, we created a dichotomous variable with a score of 1 or more identifying participants with any mobility impairment.

Activities of daily living (ADLs) were assessed by asking participants if they had difficulty performing six everyday activities because of a health or memory problem (excluding any that they expected to last less than three months). Activities included bathing or showering, preparing a hot meal and shopping for groceries. A binary (yes/no) response format was used, with scores ranging from 0 to 6 according to the number of activities with which a difficulty was reported. Scores were dichotomised to distinguish between participants reporting any ADL impairment (score of 1 or more) and those with no ADL impairment (score of 0).

Quality of life was assessed using the CASP‐19, a validated measure developed specifically to assess quality of life in early old age 14. It contains 19 items on four sub‐domains: control, autonomy, pleasure and self‐realisation. Respondents indicate their agreement with each statement, for example: ‘*my age prevents me from doing things I would like to do’,* on a 4‐point Likert scale from 0 (never) to 3 (often). Total scores ranged from 0 to 57 with higher scores indicating higher quality of life.

#### Psychological well‐being

Depressive symptoms were assessed using an 8‐item version of the Center for Epidemiologic Depression Scale 15. Respondents were asked to indicate if they had experienced depressive symptoms (e.g. restless sleep and being unhappy) over the past month using a binary (yes/no) response. Total scores ranged from 0 to 8 with higher scores indicating more depressive symptoms. Data were highly skewed, so we dichotomised scores using an established cut‐off, with a score of 4 or higher indicating the presence of depressive symptoms 16.

Life satisfaction was assessed using the Satisfaction with Life Scale 17. Respondents are asked to indicate their agreement with five statements, for example: ‘*In most ways my life is close to my ideal*’, on a 7‐point Likert scale from 0 (strongly disagree) to 6 (strongly agree). Total scores ranged from 0 to 30 with higher scores indicating greater life satisfaction. Life satisfaction was not assessed in wave 1 of ELSA, so analyses were restricted to participants with new cancer diagnoses in waves 3 to 6, with scores from waves 2 to 6 used to create means for the comparison group.

### Statistical analyses

Demographic characteristics and co‐morbidities of the two groups were compared using *t*‐tests for continuous variables and chi‐squared analyses for categorical variables. Repeated‐measures analyses of variance (ANOVAs) (continuous variables) and generalised estimating equations (categorical variables) were used to examine main effects of group (overall group differences in self‐rated health, mobility impairments, ADL impairments, quality of life, depressive symptoms and life satisfaction independent of time), main effects of time (changes in health and well‐being over time independent of group) and group‐by‐time interactions (differences in changes in health and well‐being over time between groups). Age, sex and wealth were entered as covariates for all analyses.

## Results

### Demographic characteristics

The characteristics of the 4565 participants, comprising 444 (9.7%) who received a cancer diagnosis and 4121 in the comparison group are shown in Table 1. Of the 444 cancer diagnoses, 3% (*n* = 12) were lung cancer, 20% (N=89) were breast cancer, 16% (N=69) were colorectal cancer, 21% (N=91) were skin cancer, and 41% (*n* = 183) had another cancer. Those in the cancer group were older (67.4 vs 65.0; *p* < .001), and more of them were male (49.8% vs 43.8%, *p* < .05), but the groups did not differ by wealth (*p* = .359). Compared with the comparison group, those in the cancer group were more likely to have been diagnosed with CHD (21% vs. 12%, *p* < .001), diabetes (23% vs. 14%, *p* < .001), arthritis (59% vs. 52%, *p* < .01), stroke (13% vs. 7%, *p* < .001) and chronic lung disease (14% vs. 10%, *p* < .01). There were no differences for asthma or hypertension.

**Table 1:** Demographic characteristics of sample—percentage (*n*), mean (SD)

Characteristics	Cancer group (*n* = 444)	Comparison group (*n* = 4121)	*p*
Age	67.36 (9.00)	65.04 (8.54)	<.001
Sex			
Male	49.8% (221)	43.8% (1806)	
Female	50.2% (223)	56.2% (2315)	.016
Wealth quintiles			
1 (lowest)	12.4% (55)	12.9% (531)	
2	16.9% (75)	17.8% (733)	
3	24.1% (107)	20.4% (839)	
4	20.5% (91)	23.3% (960)	
5 (highest)	26.1% (116)	25.7% (1058)	.359

### Health and quality of life

**Figure 1:**
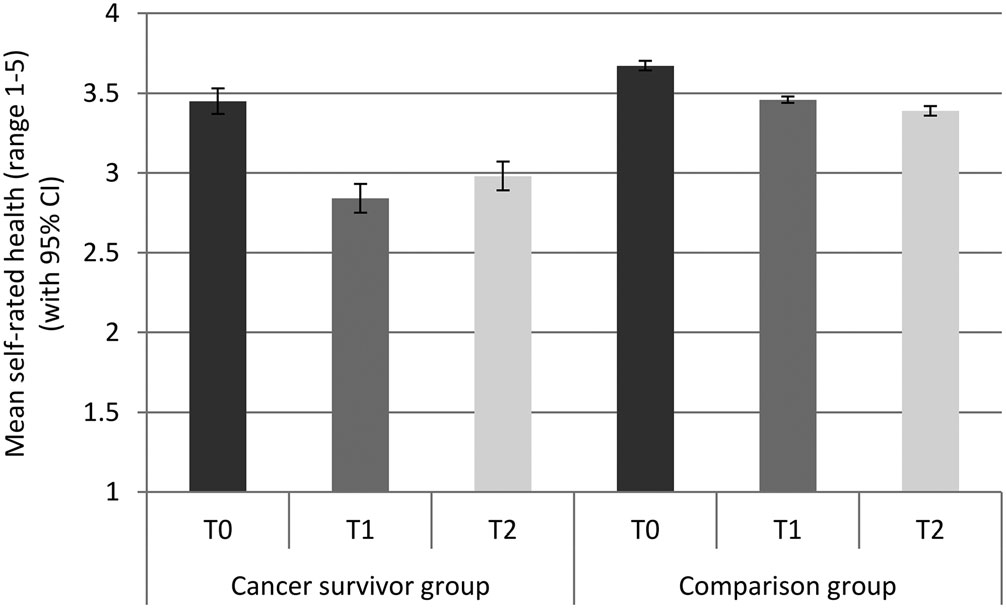
Mean self‐rated health (with 95% confidence intervals) for each group at each time point (adjusted for age, sex and wealth)

Figure 1 shows the mean self‐rated health of each group at each time. Participants in the cancer group had worse self‐rated health (*p* < .001) and overall, self‐rated health decreased over time (*p* < .001). Between T0 and T1, mean self‐rated health scores dropped from 3.45 to 2.84 in the cancer group and from 3.67 to 3.46 in the comparison group. Between T1 and T2, mean self‐rated health increased slightly from 2.84 to 2.98 in the cancer group and fell from 3.46 to 3.39 in the comparison group. The group‐by‐time interaction was significant (*p* < .001), indicating a greater decline in self‐rated health over time in the cancer group compared to the comparison group.

**Figure 2:**
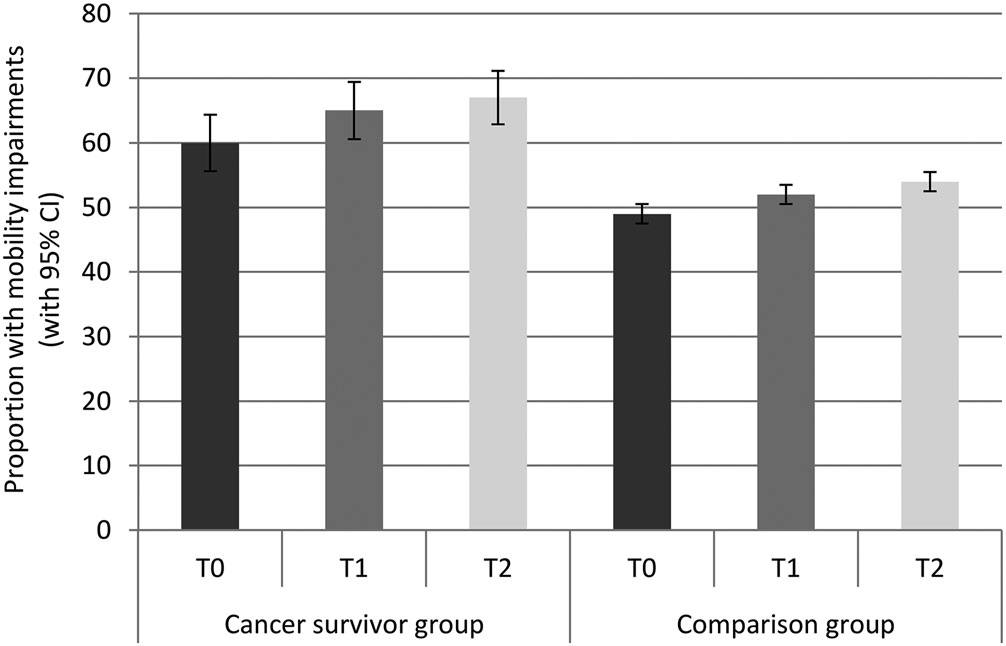
Proportion with mobility impairments (with 95% confidence intervals) in each group at each time point (adjusted for age, sex and wealth)

Figure 2 shows the proportion of participants with mobility impairments for each group at each time. A greater proportion of the cancer group had mobility impairments (*p* < .001) and overall, the proportion with mobility impairments rose over time (*p* < .001). Between T0 and T1, the proportion of participants with mobility impairments increased from 60 to 65% in the cancer group and from 49 to 52% in the comparison group. Between T1 and T2, the proportion with mobility impairments increased from 65 to 67% in the cancer group and from 52 to 54% in the comparison group. The group‐by‐time interaction was not significant (*p*=.572).

**Figure 3:**
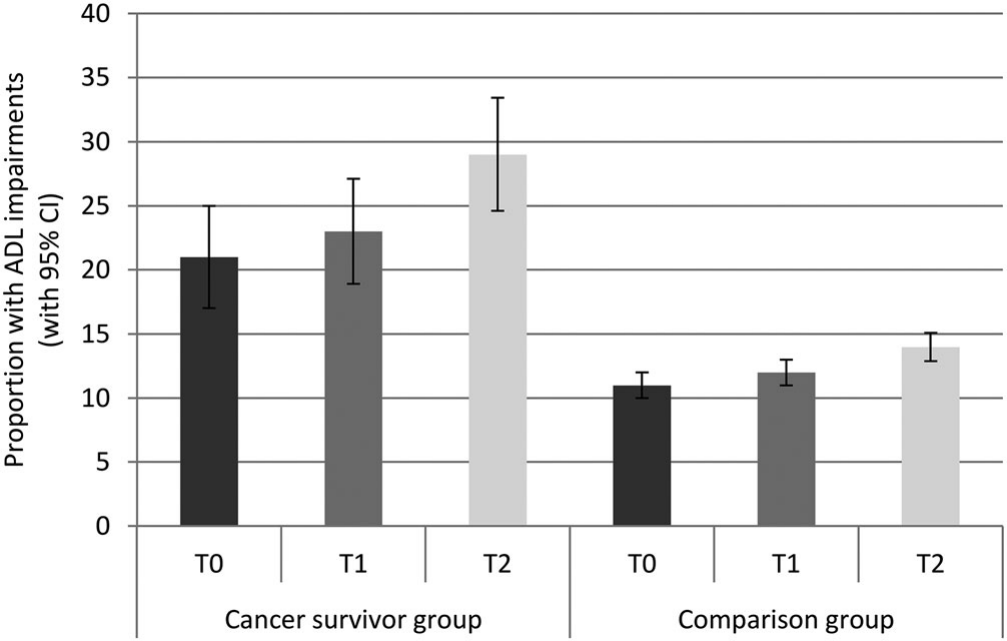
Proportion with ADL impairments (with 95% confidence intervals) in each group at each time‐point (adjusted for age, sex and wealth)

Figure 3 shows the proportion of participants with ADL impairments for each group at each time. A greater proportion of the cancer group had ADL impairments (*p* < .001). The proportion with ADL impairments increased over time (*p* < .001). Between T0 and T1, the proportion of participants with ADL impairments increased from 21 to 23% in the cancer group and from 11 to 12% in the comparison group. Between T1 and T2, the proportion with ADL impairments increased from 23 to 29% in the cancer group and from 12 to 14% in the comparison group. The group‐by‐time interaction was not significant (*p* = .330).

**Figure 4:**
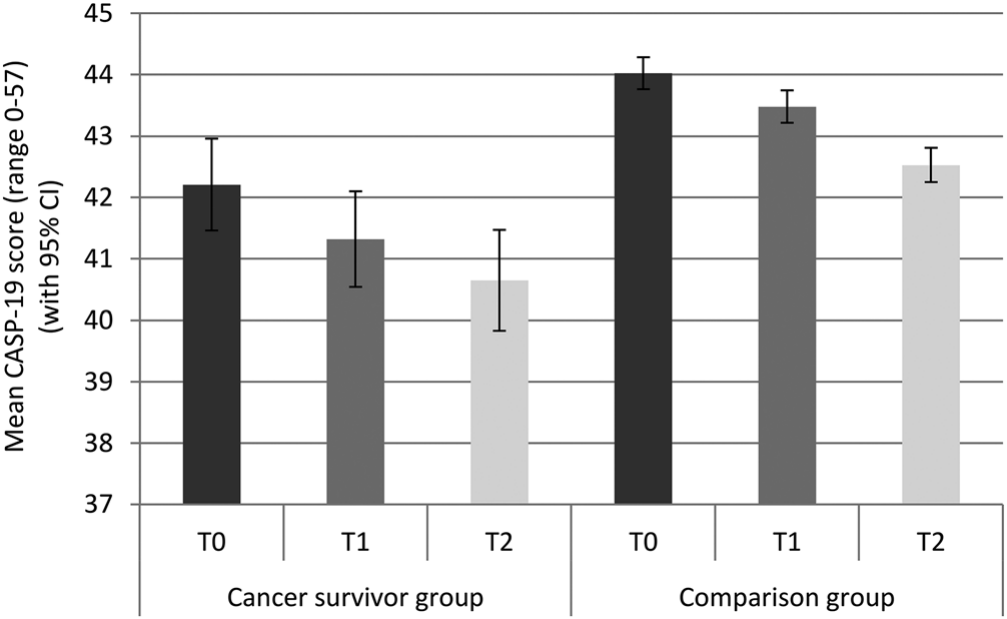
Mean quality of life (with 95% confidence intervals) for each group at each time point (adjusted for age, sex and wealth)

Figure 4 shows the mean total CASP‐19 scores for each group at each time. Those in the cancer group had significantly lower quality of life (*p* < .001), and overall, scores decreased over time (*p* < .001). Between T0 and T1, scores fell from 42.2 to 41.3 in the cancer group and from 44.0 to 43.5 in the comparison group. Between T1 and T2, scores fell from 41.3 to 40.7 in the cancer group and from 43.5 to 42.5 in the comparison group. The group‐by‐time interaction was not significant (*p* = .126).

### Psychological well‐being

**Figure 5:**
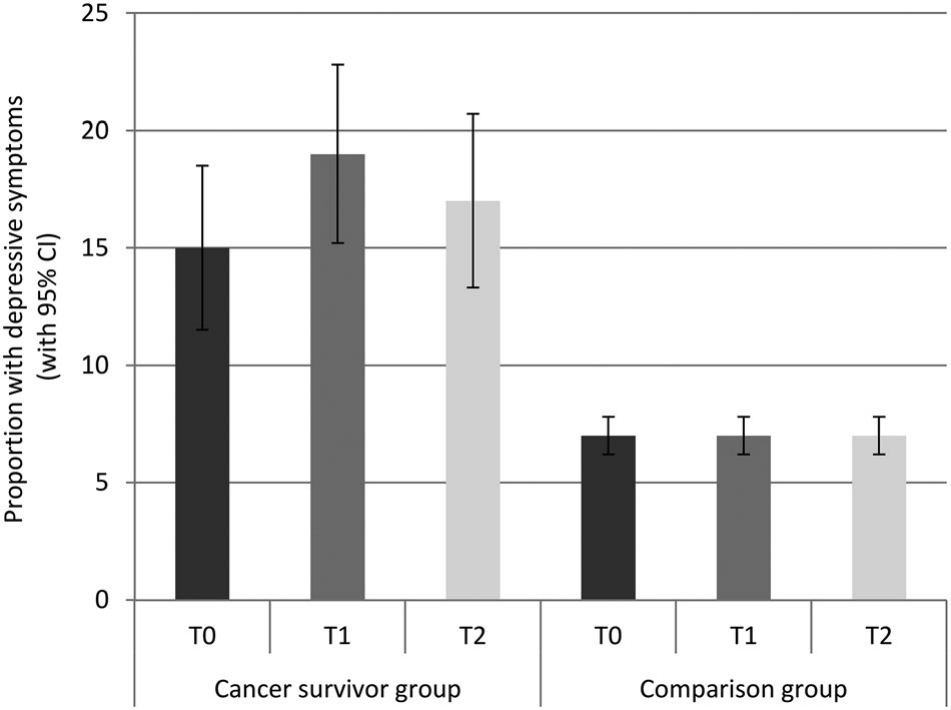
Proportion with depressive symptoms (with 95% confidence intervals) in each group at each time point (adjusted for age, sex and wealth)

Figure 5 shows the proportion of participants with depressive symptoms in each group at each time. A greater proportion of the cancer group had depressive symptoms (*p* < .001). Overall, the proportion with depressive symptoms increased and then declined over time (*p* = .027). Between T0 and T1, the proportion of participants with depressive symptoms rose from 15 to 19% in the cancer group and did not change in the comparison group (7%). Between T1 and T2, the proportion of participants with depressive symptoms dropped from 19 to 17% in the cancer group and did not change in the comparison group. This group‐by‐time interaction was not significant (*p* = .084).

**Figure 6:**
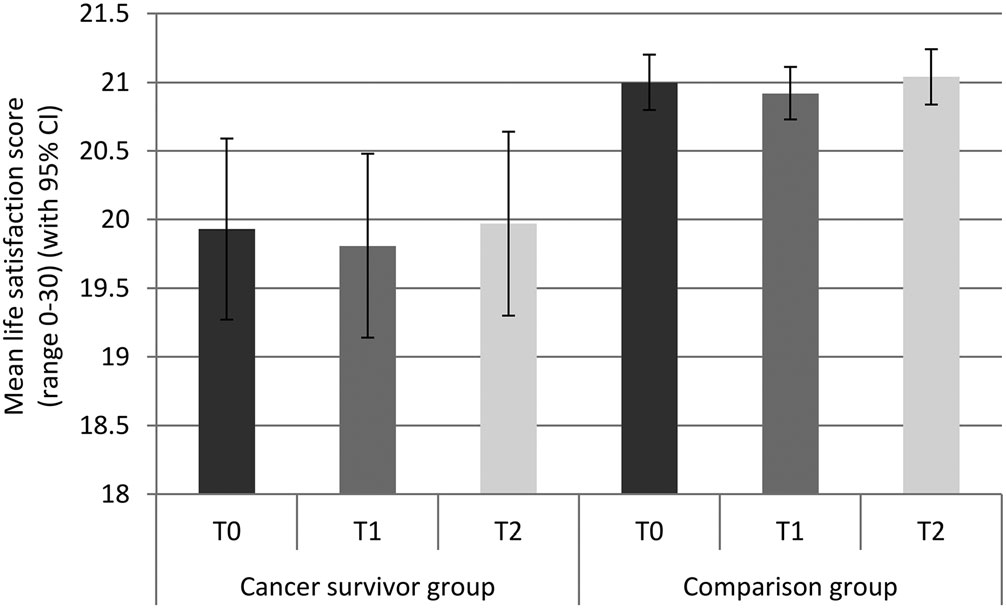
Mean life satisfaction (with 95% confidence intervals) for each group at each time point (adjusted for age, sex and wealth)

Figure 6 shows the mean total life satisfaction scores for each group at each time. Those in the cancer group had significantly lower life satisfaction (*p* < .01). Overall, life satisfaction reduced and then increased over time (*p* < .001). Between T0 and T1 scores dropped from 19.9 to 19.8 in the cancer group and from 21.0 to 20.9 in the comparison group. Between T1 and T2, scores rose from 19.8 to 20.0 in the cancer group and from 20.9 to 21.0 in the comparison group. The group‐by‐time interaction was not significant (*p* = .937).

## Discussion

This study investigated the impact of a cancer diagnosis on health and well‐being in a population‐based sample of older adults living in England. At all time points, those with a diagnosis of cancer reported poorer self‐rated health, quality of life and life satisfaction than those with no diagnosis, and a higher proportion reported depressive symptoms and impairments in mobility and ADL. In both groups, all markers of health and well‐being worsened significantly over time, with the exception of depressive symptoms. Those with a cancer diagnosis showed a greater decline in self‐rated health over time compared with the comparison group.

Cancer survivors reported being in worse health than those with no diagnosis, consistent with previous reports of poor health in this population 6, 7. This was also the case before their cancer diagnosis, which may be attributed to them experiencing potential signs and symptoms of cancer in the lead up to their diagnosis. Around the time of their diagnosis, they reported a greater reduction in their health, which is unsurprising given that they had been given a serious diagnosis. Although their self‐reported health recovered slightly a couple of years later, it did not returned to pre‐diagnosis levels, consistent with reports that physical symptoms can persist for many years following a cancer diagnosis and highlighting the impact of the long‐term effect of cancer 10.

We found that cancer survivors experienced more depressive symptoms than those without a cancer diagnosis 4. This is in line with previous studies, which have shown that as many as 58% of cancer survivors may report depressive symptoms 18. Interestingly, this was also the case before their cancer diagnosis, suggesting that impaired well‐being may begin several months or even years before the diagnosis of cancer. Future research could explore how long before a diagnosis individuals start to show such impairments. However the proportion of individuals experiencing depressive symptoms increased over time in both groups, suggesting that a cancer diagnosis does not have a particularly specific impact on the experience of depressive symptoms. However, there was a non‐significant spike in the proportion of those experiencing depressive symptoms in the cancer group around the time of diagnosis, highlighting the potential adverse effect of a cancer diagnosis. Similar to previous studies 10, the proportion of cancer survivors experiencing depressive symptoms appeared to drop slightly over time although it remained higher than both pre‐diagnosis levels and the comparison group. These findings highlight the importance of psychological assessment in this population.

A greater proportion of cancer survivors had impairments in mobility or ADL, similar to findings from cross‐sectional studies 6. Again, this was also the case before their cancer diagnosis, suggesting that cancer may begin to affect functioning even before a diagnosis is made. This may be a direct consequence of a cancer diagnosis but it could also be due to long‐standing characteristics, such as poorer health behaviours. The proportion of those experiencing ADL and mobility impairments increased across the three time points by roughly the same degree in both groups. Although this suggests that a cancer diagnosis itself did not have a substantial impact on impairments, it is consistent with a previous study that showed a decline in physical and social functioning between 5 and 8 years post‐cancer diagnosis 11.

Those who received a diagnosis of cancer had poorer quality of life and lower life satisfaction than those with no diagnosis, similar to previous studies 3, 4, 9. This was also the case at the pre‐diagnosis time point, suggesting that well‐being may be affected by cancer even before a diagnosis is made. This may be due to the undiagnosed cancer making them feel unwell and thus impacting on their psychological well‐being, although it could also be related to the same long‐standing characteristics that also cause this group to have more mobility and ADL impairments. There was a change in quality of life and life satisfaction over the three time points but this did not differ by group, suggesting that a cancer diagnosis does not adversely affect these aspects of well‐being. However, even not triggered by the cancer diagnosis, quality of life declined over time and remained worse in the cancer group, highlighting an opportunity of psychological intervention.

This study had a number of limitations. The cancer data were self‐reported, and it is possible that people may not have reported a diagnosis that was a long time ago, or they may have reported having cancer if they were diagnosed with a benign tumour. We did not have the exact date of diagnosis; which could have been any time from just after T0 or just before T1 (a range of 2 years). It is therefore possible that there may have been short‐term changes in health and well‐being that were not captured by this study. The sample size was not large enough to analyse by cancer site, and it is possible that the effects would have been different for diagnoses where prognosis was better or worse, or where treatment was more or less damaging. Our analyses only included individuals with three waves of data and as a result, those who died or elected not to take part any longer were excluded.

In conclusion, we found that cancer survivors were worse off those with no diagnosis on all aspects of health and well‐being. Interestingly, cancer survivors were more impaired in several of these domains even before their diagnosis was made. Screening for these factors around the time of a cancer diagnosis could allow for interventions to be targeted effectively and improve the health and well‐being of cancer survivors. We also found that a cancer diagnosis had an adverse effect on self‐rated health which continued to deteriorate at a greater rate than controls over time. Ensuring that people diagnosed with cancer receive appropriate psychological support could help minimise the impact of a diagnosis.

## Conflict of interest

The authors have no conflicts of interest.
